# Diagnostic Criteria during SARS Outbreak in Hong Kong

**DOI:** 10.3201/eid1006.030618

**Published:** 2004-06

**Authors:** Louis Y. Chan, Nelson Lee, Paul K.S. Chan, Alan Wu, Timothy H. Rainer, Philip K.T. Li, Hong Fung, Joseph JY Sung

**Affiliations:** * Prince of Wales Hospital, Hong Kong, Special Administrative Region (SAR);; † The Chinese University of Hong Kong, Hong Kong, SAR

**Keywords:** diagnostic criteria, SARS

**To the Editor:** A novel coronavirus caused more than 8,000 probable cases of severe acute respiratory syndrome (SARS) worldwide (*1**,**2*) during the 2003 outbreak. Before the etiologic agent was identified, the diagnosis of SARS was made according to a set of clinical-epidemiologic criteria as suggested by the Centers for Disease Control and Prevention (CDC) (*1**–**3*). These criteria remained important in the initial diagnosis and prompt isolation of patients because the overall sensitivity of initial reverse transcriptase-polymerase chain reaction (RT–PCR) testing for SARS-associated coronavirus (SARS-CoV) RNA on upper respiratory specimens ranged from approximately 60% to 70% (though sensitivity improved with a second test) (*4**,**5*). In a SARS screening clinic at the Prince of Wales emergency department, the positive predictive value (PPV) of these criteria was estimated to be 54% (95% CI 39% to 69%) (*6*). The relative importance of the clinical versus epidemiologic criteria had not been evaluated. By using paired serologic testing to determine SARS-CoV infection (*3*), we evaluated the relative importance of the clinical-epidemiologic diagnostic criteria during an outbreak.

Patients with a diagnosis of SARS, and who were admitted to one of five regional hospitals in Hong Kong for isolation and treatment from March 4 to June 6, 2003, were included in this retrospective analysis. Probable SARS case-patients were those who met the CDC clinical criteria for severe respiratory illness of unknown etiology (*3*), and met the epidemiologic criterion for exposure in either a close or a possible contact. Close contact was defined as caring for, living with, or having direct contact with body fluids of a probable SARS patient (e.g., working in the same medical ward or staying in the same household) within 10 days of initial symptoms. Because Hong Kong was the documented SARS transmission site from February 1 to July 11, 2003, a modified epidemiologic criterion of possible contact was adopted. Possible contact was defined as staying or working in the same hospital compound, or residing in the same building where case clusters of SARS had been reported, within 10 days of symptoms onset.

Laboratory testing of paired immunoglobulin (Ig) G antibody to SARS-CoV was used to determine infection (*7*). Positive serologic evidence of infection was defined as a four-fold rise in antibody titer or detection of antibody in convalescent-phase serum. Seronegativity was defined as absence of antibody in convalescent-phase serum obtained >21 days after symptom onset (*3*). Seronegativity in this defined time frame (>21 days – serum collected before July 11, 2003, and beyond 28 days) excluded the diagnosis of SARS (*3*). Samples from patients showing nonspecific fluorescent signals were considered negative for SARS-CoV infection. RT-PCR was performed on clinical specimens (respiratory, fecal) from all patients (*1**,**3**–**5*).

Demographic and laboratory parameters and history of close contact were compared between the seropositive and seronegative groups. Student *t* test was used to analyze continuous variables. A p value of <0.05 was considered statistically significant. Odds ratio (OR) and 95% confidence interval (CI) were calculated for categorical variables.

During the study period, 475 patients were hospitalized with probable SARS. One hundred patients were excluded because their serologic results were either missing (n = 37) or they died before day 21 of illness (no convalescent-phase serum, n = 63). Three hundred seventy-five patients were included in the analyses; 353 (94.1%) patients were serology-positive for SARS-CoV. Two hundred sixty-three of the 353 patients (74.5%) had a 4-fold increase in antibody titers, and 90 of the 353 patients (25.5%) had detectable antibody in either acute- or convalescent-phase serum samples (titer 80–5,120). Twenty-two patients (5.9%) had antibody titer <40 in their convalescent-phase serum samples (median = 31 days; range = 21–61 days). No clinical specimens were positive for SARS-CoV by RT-PCR. Thus, the PPV of the clinical-epidemiologic criteria for SARS in our cohort was 0.94 (95% CI 0.91–0.96).

The contact history and demographic and laboratory parameters for both seropositive and seronegative groups are depicted in the Table. The proportion of patients with a history of close contact was significantly higher in the seropositive group than in the seronegative group (91.2% vs 31.8%, OR 22.3; 95% CI 8.4–58.7). Only 8.8% of the patients with serologically confirmed results had no close contact history; 68.2% of the seronegative patients were in this category. The PPV of close contact was 0.98 (95% CI 0.96–0.99), and the PPV of possible contact was 0.67 (95% CI 0.54–0.81). Seropositive patients had a significantly lower lymphocyte count on admission compared to the seronegative patients (1.0 + 0.4 vs 1.2 + 0.8 x 10^9^/L) (p = 0.027). The PPVs for possible contact plus lymphopenia <0.8 x 10^9^/L and <1.0 x 10^9^/L were 0.76 (95% CI 0.56–0.97) and 0.72 (95% CI 0.56–0.89), respectively. Seronegative patients were older (51.2 + 24.3 vs. 40.9 + 17.2 years), were less likely to be healthcare workers (90.9% vs. 45.3%), had their venue of contact in the community (63.6% vs. 17.8%), and had a higher total leukocyte count on admission (9.4 + 7.4 vs. 6.2 + 3.2 x 10^9^/L). No differences were found in the lactate dehydrogenase, activated partial thromboplastin time, creatinine phosphokinase, and alanine-aminotransferase levels between the two groups.

**Table:** SARS contact history and demographic and initial laboratory parameters in seropositive and seronegative patients

	Seropositive patients, n = 353 (%)	Seronegative patients, n = 22 (%)	p value or OR (95% CI)^a^
Demographic data			
Age	40.9 + 17.2	51.2 + 24.3	0.008
Healthcare workers (HCW)	193 (54.7)	2 (9.1)	12.1 (2.8 to 52.4)
Non-HCW	160 (45.3)	20 (90.9)	
Laboratory parameters on admission			
Total leukocyte count (x 10^9^/L)	6.2 + 3.2	9.4 + 7.4	<0.001
Lymphocyte count (x 10^9^/L)	1.0 + 0.4	1.2 + 0.8	0.027
Level of contact			
Definite close contact	322 (91.2)	7 (31.8)	22.3 (8.4 to 58.7)
Possible contact	31 (8.8)	15 (68.2)	
Possible contact plus lymphopenia			
Lymphocyte < 0.8 x 10^9^/L	13 (76.5)	4 (23.5)	
Lymphocyte < 1.0 x 10^9^/L	21 (72.4)	8 (27.6)	
Venue of contact			
Hospital	290 (82.2)	8 (36.4)	8.1 (3.2 to 20.0)
Community	63 (17.8)	14 (63.6)	

^a^OR, odds ratio; CI, confidence interval.

Fifteen of the 22 seronegative patients responded to antibiotics (*8*); five died of comorbid illnesses (one of carcinoma of lung, one of metastatic carcinoma of prostate, two of chronic pulmonary diseases, and one of congestive heart failure), and two died of bacterial pneumonia. In four patients, bacterial pathogens were identified (one methicillin-resistant *Staphylococcus aureus*, two *Stenotrophomonas maltophilia*, and one *Pseudomonas aeruginosa*). Also, 15 (68.2%) of the patients had coexisting medical conditions: three had congestive heart failure, four had chronic pulmonary diseases, two had chronic renal failures, two had advanced malignancies, two had diabetes mellitus, and two had Parkinson's disease.

Our findings showed that 5.9% of cases defined as probable SARS on the basis of clinical-epidemiologic criteria had no serologic evidence of coronavirus infection. This set of criteria was associated with a PPV as high as 0.94 in a local outbreak. The PPV of the CDC epidemiologic criterion of close contact was higher (0.98). The PPV of possible contact was 0.67, but when applied with lymphopenia, the PPV became higher. Our analysis illustrated that a history of close contact with patients with SARS-CoV infection is of major importance when diagnosing such infection. This finding supports the hypothesis that SARS-CoV is transmitted through respiratory droplets and physical contact with a patient's body fluids. Although not specific, lymphopenia and its subsequent progress was highly prevalent among SARS patients (*8**–**10*). Clinicians are now advised by the World Health Organization that hematologic deviations (e.g., lymphopenia) should be considered in SARS evaluations (*1*).

Our study was limited by sample size and its retrospective status. Nonetheless, we demonstrated the accuracy of diagnostic criteria in an outbreak and the importance of epidemiologic criteria. Further studies are needed to evaluate the diagnostic accuracy of these criteria in a nonoutbreak situation when the case prevalence is low.
